# Treatment adherence and health outcomes in patients with bronchiectasis

**DOI:** 10.1186/1471-2466-14-107

**Published:** 2014-07-01

**Authors:** Amanda R McCullough, Michael M Tunney, Alexandra L Quittner, J Stuart Elborn, Judy M Bradley, Carmel M Hughes

**Affiliations:** 1 Clinical & Practice Research Group, School of Pharmacy, Queen’s University Belfast, 97 Lisburn Road, Belfast BT9 7BL, UK; 2 Department of Psychology, University of Miami, Coral Gables, FL, USA; 3 Centre for Infection and Immunity, School of Medicine, Dentistry and Biomedical Sciences, Queen’s University Belfast, Belfast, UK; 4 Centre for Health and Rehabilitation Technologies (CHaRT), Institute of Nursing and Health Research, University of Ulster, Jordanstown, UK

**Keywords:** Patient adherence, Bronchiectasis, Physical therapy, Drug therapy, Quality of Life Questionnaire-Bronchiectasis

## Abstract

**Background:**

We aimed to determine adherence to inhaled antibiotics, other respiratory medicines and airway clearance and to determine the association between adherence to these treatments and health outcomes (pulmonary exacerbations, lung function and Quality of Life Questionnaire-Bronchiectasis [QOL-B]) in bronchiectasis after 12 months.

**Methods:**

Patients with bronchiectasis prescribed inhaled antibiotics for *Pseudomonas aeruginosa* infection were recruited into a one-year study. Participants were categorised as “adherent” to medication (medication possession ratio ≥80% using prescription data) or airway clearance (score ≥80% in the Modified Self-Reported Medication-Taking Scale). Pulmonary exacerbations were defined as treatment with a new course of oral or intravenous antibiotics over the one-year study. Spirometry and QOL-B were completed at baseline and 12 months. Associations between adherence to treatment and pulmonary exacerbations, lung function and QOL-B were determined by regression analyses.

**Results:**

Seventy-five participants were recruited. Thirty-five (53%), 39 (53%) and 31 (41%) participants were adherent to inhaled antibiotics, other respiratory medicines, and airway clearance, respectively. Twelve (16%) participants were adherent to all treatments. Participants who were adherent to inhaled antibiotics had significantly fewer exacerbations compared to non-adherent participants (2.6 vs 4, p = 0.00) and adherence to inhaled antibiotics was independently associated with having fewer pulmonary exacerbations (regression co-efficient = -0.51, 95% CI [-0.81,-0.21], p < 0.001). Adherence to airway clearance was associated with lower QOL-B Treatment Burden (regression co-efficient = -15.46, 95% CI [-26.54, -4.37], p < 0.01) and Respiratory Symptoms domain scores (regression co-efficient = -10.77, 95% CI [-21.45; -0.09], p < 0.05). There were no associations between adherence to other respiratory medicines and any of the outcomes tested. Adherence to treatment was not associated with FEV_1_ % predicted.

**Conclusions:**

Treatment adherence is low in bronchiectasis and affects important health outcomes including pulmonary exacerbations. Adherence should be measured as part of bronchiectasis management and future research should evaluate bronchiectasis-specific adherence strategies.

## Background

Non-cystic fibrosis (CF) bronchiectasis is an under-researched condition with no licensed treatment therapies [1]. Approximately 20% of the bronchiectasis population are infected with *Pseudomonas aeruginosa*[2], which is known to be associated with a more rapid decline in lung function [3] and poorer health-related quality of life (HRQoL) [4]. In line with published guidelines, inhaled antibiotics along with other inhaled and oral medications are commonly prescribed ‘off-label’ for these patients, with the aim of reducing pulmonary exacerbations, maintaining lung function and improving HRQoL [5]. Airway clearance is also routinely prescribed for these patients leading to a complex and burdensome treatment regimen.

Adherence to similar medications is consistently reported to be low in asthma, chronic obstructive pulmonary disease (COPD) and CF, with approximately 50% of patients reported to be non-adherent [6-8]. Adherence to airway clearance is even lower than for medications, with up to 70% of patients with CF non-adherent to this treatment [9]. In bronchiectasis, a study exploring patients’ perspectives on self-management reported that patients altered their adherence to treatments [10]. A single study measured adherence in 22 patients with bronchiectasis prescribed colistin delivered via I-neb^®^ adaptive aerosol delivery (Praxis Pharmaceuticals) or Pari LC^®^ Plus and reported high levels of adherence, with 73% of participants categorised as adherent [11]. However, this study had a small sample size, was retrospective, measured adherence to Pari LC^®^ Plus via self-report and did not determine if adherence was associated with health outcomes.

Studies in asthma, COPD and CF demonstrate that low adherence to treatment can negatively impact on key health outcomes including pulmonary exacerbations [8,12], HRQoL [6,13] as well as healthcare costs [14,15]. The first randomised controlled trial investigating the effectiveness of colistin (Promixin^®^) in bronchiectasis has shown that it is effective in delaying time to first pulmonary exacerbation and improving HRQoL when patients are adherent i.e. inhale between 81–92.1% of prescribed medication [16]. Thus, rates of adherence to treatment may affect important health outcomes such as pulmonary exacerbations, lung function and HRQoL in bronchiectasis. Rates of treatment adherence and the effect of treatment adherence on these health outcomes in bronchiectasis are not known. With large-scale studies of new and potentially expensive therapies that have been tailored for use in patients with bronchiectasis infected with *P. aeruginosa* underway [16,17], it is important to determine treatment adherence in this population to ensure that adherence is maximised prior to the prescription of these new therapies.

Thus, the primary aim of this one-year study was to determine rates of treatment adherence (inhaled antibiotics, other respiratory medicines and airway clearance) in patients with bronchiectasis infected with *P. aeruginosa* over the year-long study. The secondary aim was to determine the association between adherence to treatment and health outcomes (pulmonary exacerbations, lung function and Quality of Life Questionnaire-Bronchiectasis [QOL-B]) in this population after 12 months. It was hypothesised that adherence would be low and patients who were adherent to inhaled antibiotics would have fewer pulmonary exacerbations.

## Methods

### Participants

Participants were recruited from the Regional Respiratory Centre at Belfast City Hospital and from seven secondary care hospitals across Northern Ireland (June 2010-August 2011). Adult patients with a confirmed diagnosis of bronchiectasis by high-resolution computed tomography, who commenced inhaled colistin or tobramycin by nebuliser ≥6 weeks prior to recruitment and had a positive sputum culture for *P. aeruginosa* prior to starting inhaled antibiotics, were recruited (Additional file 1 contains a copy of the patient information sheet). Participants had to be clinically stable (≥2 weeks post completion of treatment for a pulmonary exacerbation) prior to data collection. Ethical approval was obtained from the Office for Research Ethics Northern Ireland (10/NIR03/17). All participants provided written informed consent. As part of usual care in all centres, participants had received training from a specialist nurse and/or physiotherapist on how to use nebulisers and inhalers. All participants received education about how to complete airway clearance from a specialist physiotherapist. All participants were routinely followed-up by respiratory consultants and/or nursing/physiotherapy.

### Study design

A one-year study was conducted (June 2010-August 2012). Data were collected at baseline followed by 3 or 6 monthly intervals.

A modified version of the Self-reported Medication-taking Scale [18] including an additional question [19] (e-Table 1, Additional file 1) was interviewer-administered in person (baseline, 6 and 12 months) or via telephone (3 and 9 months) for each treatment: inhaled antibiotics, other respiratory medicines (e.g. inhaled corticosteroids or oral azithromycin) and airway clearance. Each questionnaire contained five questions which were answered either ‘yes’ (scored as 0) or ‘no’ (scored as 1) (e-Table 1). The maximum score was of 5, with higher scores indicating higher adherence. Participants were categorised as adherent to each treatment by scoring ≥4 (≥80%) on this scale at all data collection points. There is no consensus in the literature about an appropriate cut-off for adherence as thresholds for treatment efficacy are not known. However, landmark bronchiectasis clinical trials consistently use 80% as their threshold for adherence [17,20] and the 80% cut-off has been shown to offer the optimal balance between specificity and sensitivity for self-reported, prescription refill and electronic pillbox adherence measures [19].

**Table 1:** Baseline characteristics of study participants (n = 75)

**Characteristic**		**Result**
Age, yr		64 ± 8
Gender, M/F		24 (32)/51 (68)
Occupational status, n (%)	Employed/self-employed	18 (24)
	Retired	57 (76)
Level of education, n (%)	Less than high school	14 (19)
	High school	54 (72)
	University	7 (9)
Marital status, n (%)	Married or with a partner	57 (76)
	Not married or not with a partner	18 (24)
FEV_1_, L		1.34 ± 0.6
FEV_1,_% predicted		61 ± 25
Smoking status, n (%)	Never	53 (71%)
	Current or ex	22 (29%)
Aetiology, n (%)	Post-infection	32 (43%)
	Idiopathic	16 (21%)
	Rheumatoid arthritis	8 (11%)
	COPD	5 (7%)
	Asthma	3 (4%)
	Other	11 (15%)
Prescribed medicines		12 ± 5
Inhaled antibiotics, n (%)	Colistin	64 (85%)
	Tobramycin	11 (15%)
Other respiratory medicines, n (%)		
Oral medicines	Azithromycin	38 (51%)
	Oral steroids	19 (25%)
	Mucolytics	10 (13%)
	Leukotriene receptor antagonists	8 (11%)
	Theophylline	7 (9%)
	Co-trimoxazole	1 (1%)
	Salbutamol	1 (1%)
Inhaled medicines	Short-acting beta_2_ agonists	67 (89%)
	Inhaled corticosteroids	65 (87%)
	Antimuscarinics	31 (41%)
	Long-acting beta_2_ agonists	2 (3%)
Nebulised medicines	Short-acting beta_2_ agonists	55 (73%)
	Isotonic saline	25 (33%)
	Ipratropium bromide with salbutamol	3 (4%)
	Hypertonic saline	1 (1%)
	Budesonide	1 (1%)
Airway clearance	Active cycle of breathing technique	39 (53%)
	Acapella^®^	45 (61%)
QOL-B	Physical functioning	31 ± 26
	Role functioning	45 ± 28
	Vitality functioning	37 ± 20
	Social functioning	42 ± 26
	Emotional functioning	73 ± 21
	Treatment burden	56 ± 20
	Health perceptions	39 ± 19
	Respiratory symptoms	53 ± 21

Results are presented as mean ± SD or n (%).

COPD: chronic obstructive pulmonary disease.

QOL-B: Quality of Life Questionnaire-Bronchiectasis; scores range 0–100, higher scores indicate better quality of life.

Data on prescribed medications including dosages were extracted from medical records and provided by patients on a 6 monthly basis and used to maintain an up-to-date list of prescribed medication. Participants provided us with contact details for all of the community pharmacies who dispensed their medications for bronchiectasis. In a minority of cases, participants obtained their inhaled antibiotics from a hospital pharmacy (n = 12). The researcher contacted participants’ community and hospital pharmacies on a 6 monthly basis to obtain a list of dispensed medications for the previous 6 months. All pharmacies contacted provided a full list of dispensed medication for the duration of the participant’s inclusion in the study i.e. from baseline until each participant completed the study at one year. We did not collect data on medication carryover pre-baseline or leftover at the end of the study period [8,14].

Medication possession ratios (MPRs) were calculated for inhaled antibiotics and other respiratory medicines from the medication data described above, using the number of days’ supply divided by the number of days medication was prescribed in the study (from baseline until each participant completed the study at one year) and multiplied by 100 [8,14,21]. MPR was calculated over the year rather than 6 monthly to reduce the effect of any carryover/left-over as this is likely to average out over this time [22] and to reduce the effect that seasonal variation in symptoms might have on adherence and health outcomes. Participants were expected to be in the study for 365 days with most participants completing within one week of this end date; however, this varied slightly due to patients experiencing pulmonary exacerbations which delayed final data collection. We did not exclude days of hospital admission as participants used their own medications whilst in hospital (standard practice in the included hospitals). MPR was calculated with and without removing hospital admission days and this did not result in a change of adherence categorisation for any participant. Therefore, the MPR used in this analysis was calculated based on the assumption that participants used their own medications in hospital to reduce the risk of over-estimating adherence. New medications commenced in hospital were provided by the hospital during stays and issued from community pharmacies on discharge. To accurately calculate the total number of days in the study for a new medication which was commenced in hospital, the number of days between the start and end date of the medication was calculated and the number of days supplied by the hospital was subtracted from this.

When participants were prescribed oral or intravenous (IV) antibiotics, some healthcare professionals advised participants to stop their inhaled antibiotics and/or azithromycin during this time. Therefore, depending on local policy, when calculating MPR for inhaled antibiotics and azithromycin, the number of days of IV and/or oral antibiotic usage was deducted from the number of days medication was prescribed in the study.

For adherence to other respiratory medicines, MPR was calculated for each individual medication as described above and then averaged for each participant to give a composite MPR for ‘other respiratory medicines’ [8,14]. MPR for ‘other respiratory medicines’ did not include salbutamol nebulisers or inhalers. Participants who had an MPR ≥80% were categorised as adherent for inhaled antibiotics and other respiratory medicines [8]*.*

A pulmonary exacerbation was defined as a new administration of oral or intravenous (IV) antibiotics for a respiratory infection [23]. Participants kept a record of oral and IV antibiotic usage and hospital admissions for bronchiectasis for the duration of the study and these were cross-referenced with participant medical records and prescription data. To take account of potential clustering of pulmonary exacerbations [24], antibiotics administered within 14 days of the end of a previous course of antibiotics were considered to be a continuation of the previous pulmonary exacerbation and not classified as a new pulmonary exacerbation [25]. This cut-off for exacerbations reflects data in COPD which demonstrates that it takes 14 days to recover from an exacerbation [26]. No similar data exist for bronchiectasis.

Spirometry was performed according to accepted guidelines at baseline and 12 months [27]. Participants self-completed the Quality of Life Questionnaire-Bronchiectasis (QOL-B) (version 2.0, 2008) at baseline and 12 months [28]. This version contained 45 questions. Further revisions were made by the authors (version 2.1, 2010), which removed 10 questions that patients did not perceive as relevant and added one question ‘I am worried about being exposed to others who are sick’ (scored on the Social Functioning domain). The 10 questions that were removed were not scored and the wording of the remaining questions between version 2.0 (2008) and version 2.1 (2010) was unchanged. The scoring of the questionnaire allows for one missing response in each domain; therefore, the addition of a new question did not impact the scoring for the Social Functioning domain for those who had already completed the previous version at baseline (n = 48). Version 2.1 (2010) was used for all other study time points. Standardized scores were calculated for the 8 scales of the QOL-B (Physical Functioning, Role Functioning, Vitality, Social Functioning, Emotional Functioning, Treatment Burden, Health Perceptions, Respiratory Symptoms), ranging from 0–100 for each domain; higher scores indicate better HRQoL. No total score is calculated. Psychometric properties of the QOL-B are discussed in Additional file 1.

### Statistical analysis

Assuming that adherence to inhaled medication was low in this patient group, in order to detect a difference of 2 exacerbations at 80% power and 5% significance, approximately 100 participants needed to be recruited, assuming that 80% were non-adherent and 20% were adherent. This study was conducted across 8 sites to maximise recruitment; despite this, there were insufficient numbers of eligible participants to meet the recruitment target within the study timeframe. However, the study was not underpowered to detect a difference in the frequency of pulmonary exacerbations (see details in the Discussion).

Data were included up until completion or withdrawal from the study and were entered into IBM SPSS for Windows, version 19 (IBM Corp, Armonk, NY). Between-group differences in adherence were calculated for pulmonary exacerbations, lung function at 12 months and QOL-B at 12 months using t tests. These analyses were completed using MPR for medication adherence and self-report for airway clearance adherence. A Poisson regression model with adherence to inhaled antibiotics, other respiratory medicines and airway clearance entered as independent variables and pulmonary exacerbations as the dependent variable was completed. A standard linear regression model with adherence to inhaled antibiotics, other respiratory medicines and airway clearance entered as independent variables was completed for each of the following outcomes: 12 month FEV_1_ % predicted, 12 month QOL-B Treatment Burden and Respiratory Symptoms domains. We did not complete regression models to determine associations between adherence and all eight QOL-B domains to limit the chance of type 1 error. We chose QOL-B Treatment Burden and Respiratory Symptoms as Treatment Burden is important for adherence in CF [9] and QOL-B Respiratory Symptoms is a recognised end-point for bronchiectasis clinical trials [29]. All models were adjusted for age, gender and baseline FEV_1_ % predicted. If any of the adherence groups in any of the models were significant or borderline significant (p < 0.1), we re-ran the models with that one omitted to check for ‘masking’ of effects of the other two groups.

## Results

### Study participants

**Figure 1:**
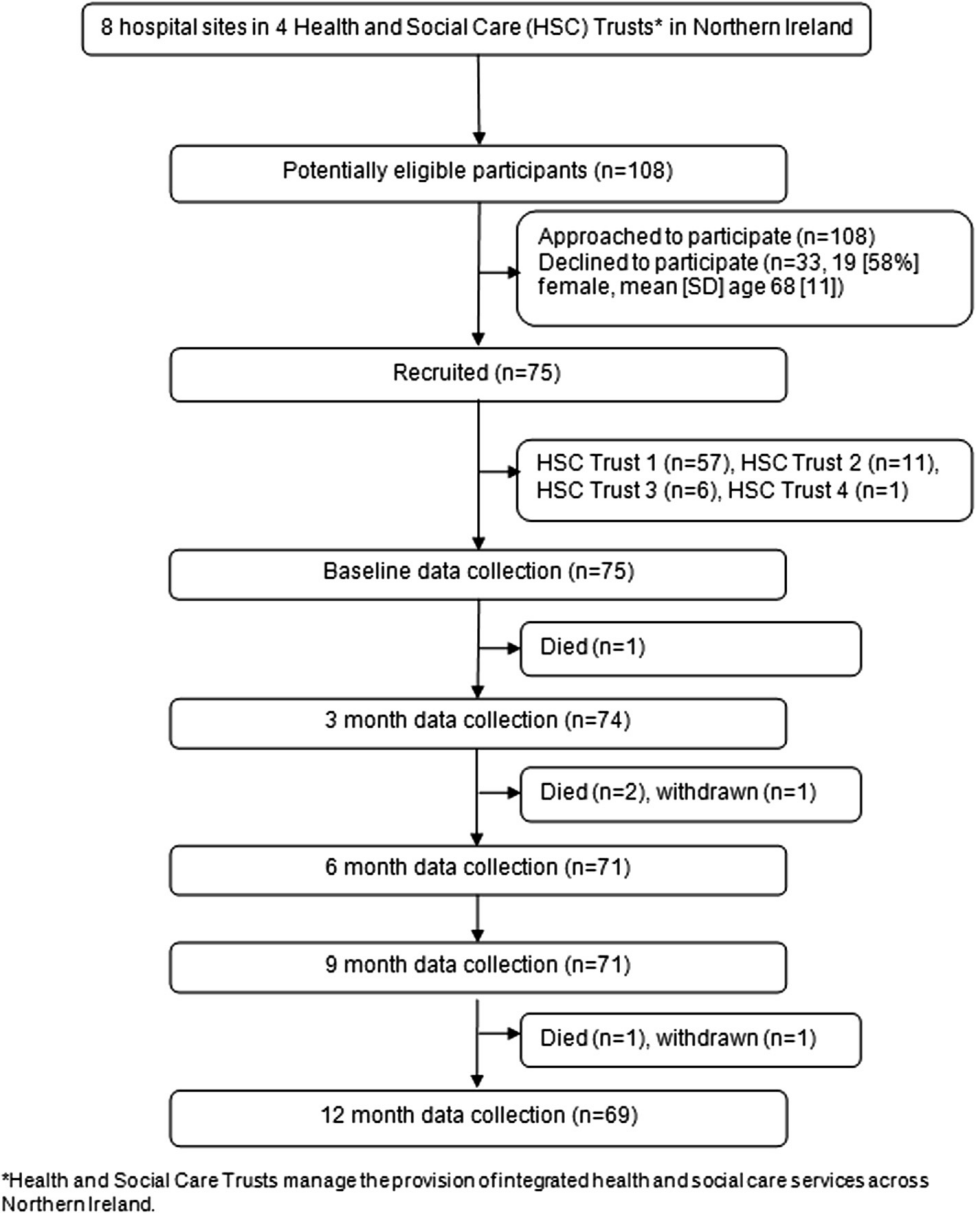
Flow of participants through the study from recruitment to completion.

Seventy-five of 108 (69%) potentially eligible participants were recruited, with 69 completing the one-year study (Figure 1). Participants were in the study for a mean ± SD of 355 ± 72 days. Participants’ baseline demographic, disease-specific, medication, airway clearance and QOL-B data are shown in Table 1.

### Adherence to treatment

More participants self-reported adherence to inhaled antibiotics (50/75 participants [67%]) and other respiratory medicines (45/74 participants [61%]) than was recorded using MPRs (inhaled antibiotics: 35/66 [53%] and other respiratory medicines: 39/73 [53%]) (Table 2). Airway clearance had the lowest rate of adherence (self-report), with 31/75 (41%) participants categorised as adherent. This was significantly lower than adherence to inhaled antibiotics (p = 0.00) and other respiratory medicines (p = 0.01). Overall, only 12 (16%) participants were categorised as adherent to all prescribed treatments and 16 (21%) were categorised as non-adherent to all treatments.

**Table 2:** Comparison between adherence groups for health outcomes measured during the one-year study

	**Inhaled antibiotics**			**Other respiratory medicines**			**Airway clearance**		
	**(n = 61**^**a**^**)**			**(n = 68**^**b**^**)**			**(n = 69**^**c**^**)**		
	Adherent (n = 32)	Non-adherent (n = 29)	p	Adherent (n = 37)	Non-adherent (n = 31)	p	Adherent (n = 27)	Non-adherent (n = 42)	p
Pulmonary exacerbations^d^	2.6 ± 2	4.0 ± 2	0.00*	3.1 ± 2.2	3.3 ± 2.3	0.75	3.1 ± 2.2	3.2 ± 2.2	0.84
FEV_1_, L at 12 months	1.36 ± 0.54	1.29 ± 0.51	0.61	1.35 ± 0.59	1.35 ± 0.50	0.97	1.29 ± 0.44	1.41 ± 0.61	0.40
FEV_1_,% predicted at 12 months	65 ± 28	56 ± 23	0.22	63 ± 30	63 ± 22	0.92	65 ± 23	62 ± 29	0.60
QOL-B at 12 months									
Physical Functioning	33 ± 28	31 ± 24	0.77	29 ± 26	39 ± 28	0.13	42 ± 28	29 ± 26	0.05*
Role Functioning	48 ± 27	45 ± 21	0.63	44 ± 24	55 ± 24	0.06	48 ± 23	50 ± 26	0.79
Vitality	39 ± 22	39 ± 20	0.92	40 ± 20	40 ± 22	0.98	39 ± 23	40 ± 20	0.73
Social Functioning	42 ± 28	41 ± 22	0.89	39 ± 26	43 ± 25	0.46	37 ± 22	44 ± 27	0.25
Emotional Functioning	73 ± 21	76 ± 18	0.60	74 ± 20	76 ± 20	0.69	73 ± 23	76 ± 18	0.49
Treatment Burden	59 ± 22	57 ± 21	0.83	61 ± 25	59 ± 20	0.66	54 ± 22	64 ± 22	0.07
Health Perceptions	40 ± 18	39 ± 13	0.80	40 ± 18	42 ± 16	0.71	40 ± 19	41 ± 16	0.79
Respiratory Symptoms	61 ± 21	51 ± 20	0.06	60 ± 22	53 ± 20	0.15	54 ± 20	58 ± 22	0.42

^a^n = 61: 6 withdrawals, 8 without adequate adherence data.

^b^n = 68: 6 withdrawals, 1 participant not prescribed treatment.

^c^n = 69: 6 withdrawals.

^d^inhaled antibiotics: n = 66: 1 participant withdrawal, 8 participants with insufficient data to calculate adherence; other respiratory medicines n = 73: 1 participant withdrawal, 1 not prescribed; airway clearance n = 74: 1 participant withdrawal.

*p < 0.05.

Results are presented as mean ± SD, median (IQR) or percentage.

FEV_1_: forced expiratory volume in one second.

QOL-B: Quality of Life Questionnaire-Bronchiectasis; scores range 0–100, higher scores indicate better quality of life.

### Health outcomes

Participants had a mean ± SD 3.2 ± 2 pulmonary exacerbations during the study. Mean ± SD FEV_1_ (1.36 L ± 0.55) and FEV_1_, % predicted (63% ± 26) at 12 months were not significantly different from baseline. Mean ± SD 12 month QOL-B Physical Functioning (34 ± 27), Role Functioning (49 ± 25), Vitality (40 ± 21), Social Functioning (41 ± 25), Emotional Functioning (75 ± 20), Treatment Burden (60 ± 22), Health Perceptions (41 ± 17), Respiratory Symptoms (57 ± 21) scores were not significantly different from baseline.

### Treatment adherence and health outcomes

Differences in health outcomes between adherence groups are summarised in Table 2. Participants who were adherent to inhaled antibiotics had significantly fewer pulmonary exacerbations and participants who were adherent to airway clearance had significantly higher QOL-B Physical Functioning scores (Table 2). No other significant differences between those who were adherent and non-adherent were noted.

Regression models for adherence and health outcomes are shown in Tables 3, 4, 5 and 6. Adherence to inhaled antibiotics was associated with having fewer pulmonary exacerbations (regression co-efficient = -0.51, 95% CI [-0.81,-0.21], p < 0.001). Adherence to other respiratory medicines and airway clearance were not associated with pulmonary exacerbations. Adherence to treatment was not associated with FEV_1_ % predicted. Adherence to airway clearance was associated with having a lower QOL-B Treatment Burden score i.e. higher treatment burden (regression co-efficient = -15.46, 95% CI [-26.54, -4.37], p < 0.01). Adherence to inhaled antibiotics and other respiratory medicines were not associated with QOL-B Treatment Burden score. Adherence to airway clearance was associated with lower Respiratory Symptoms domains scores i.e. more problematic symptoms (regression co-efficient = -10.77, 95% CI [-21.45; -0.09], p < 0.05). Adherence to inhaled antibiotics and other respiratory medicines were not associated with QOL-B Respiratory Symptoms score.

**Table 3:** **Poisson regression model for the association between adherence and pulmonary exacerbations**^**a**^

**Independent variables**	**Estimated co-efficient (SE)**	**95% CI**
Adherence to inhaled antibiotics^b^	-0.51*** (0.15)	[-0.81; -0.21]
Adherence to other respiratory medicines^b^	0.06 (0.15)	[-0.24; 0.36]
Adherence to airway clearance^b^	0.04 (0.15)	[-0.25; 0.33]
Age	-0.01 (0.01)	[-0.02; 0.01]
Gender^c^	0.17 (0.15)	[-0.14; 0.47]
Baseline FEV_1_ % predicted	0.01 (0.00)	[0.00; 0.01]

^a^n = 65: 1 participant withdrawal, 8 participants with insufficient data to calculate adherence to inhaled antibiotics and 1 participant not prescribed other respiratory medicines.

^b^Adherent = 1, non-adherent = 0.

^c^Male = 1, female = 0.

***p < 0.001.

**Table 4:** **Linear regression model for the association between adherence and FEV**_**1**_** % predicted**^**a**^

**Independent variables**	**Estimated co-efficient (SE)**	**95% CI**
Adherence to inhaled antibiotics^b^	-2.03 (2.57)	[-7.06; 3.00]
Adherence to other respiratory medicines^b^	4.43 (2.57)	[-0.61; 9.47]
Adherence to airway clearance^b^	-2.32 (2.47)	[-7.16; 2.51]
Age	-0.11 (0.15)	[-0.40; 0.17]
Gender^c^	-5.05 (2.64)	[-10.23; 0.13]
Baseline FEV_1_ % predicted	1.04*** (0.05)	[0.94; 1.15]

^a^n = 60: 6 participant withdrawals,, 8 participants with insufficient data to calculate adherence to inhaled antibiotics and 1 participant not prescribed other respiratory medicines.

^b^Adherent = 1, non-adherent = 0.

^c^Male = 1, female = 0.

***p < 0.001.

Adjusted R^2^ = 0.07.

**Table 5:** **Linear regression model for the association between adherence and 12 month QOL-B Treatment Burden**^**a**^

**Independent variables**	**Estimated co-efficient (SE)**	**95% CI**
Adherence to inhaled antibiotics^b^	-0.01 (5.88)	[-11.53; 11.51]
Adherence to other respiratory medicines^b^	-4.22 (5.89)	[-15.77; 7.34]
Adherence to airway clearance^b^	-15.46** (5.66)	[-26.54; -4.37]
Age	0.79* (0.33)	[0.14; 1.45]
Gender^c^	10.87 (6.05)	[-0.72; 24.34]
Baseline FEV_1_% predicted	0.16 (0.12)	[-0.09; 0.40]

^a^n = 60: 6 participant withdrawals, 8 participants with insufficient data to calculate adherence to inhaled antibiotics and 1 participant not prescribed other respiratory medicines.

^b^Adherent = 1, non-adherent = 0.

^c^Male = 1, female = 0.

**p < 0.01, *p < 0.05.

Adjusted R^2^ = 0.13.

**Table 6:** **Linear regression model for the association between adherence and 12 months QOL-B respiratory symptoms**^**a**^

**Independent variables**	**Estimated co-efficient (SE)**	**95% CI**
Adherence to inhaled antibiotics^b^	8.13 (5.66)	[-2.96; 19.22]
Adherence to other respiratory medicines^b^	-1.94 (5.68)	[-13.06; 9.19]
Adherence to airway clearance^b^	-10.77* (5.45)	[-21.45; -0.09]
Age	0.92** (0.32)	[0.29; 1.55]
Gender^c^	7.74 (5.83)	[-3.68; 19.17]
Baseline FEV_1_% predicted	0.07 (0.12)	[-0.16; 0.31]

^a^n = 60: 6 participant withdrawals,, 8 participants with insufficient data to calculate adherence to inhaled antibiotics and 1 participant not prescribed other respiratory medicines.

^b^Adherent = 1, non-adherent = 0.

^c^Male = 1, female = 0.

**p < 0.01, *p < 0.05.

Adjusted R^2^ = 0.16.

## Discussion

In this one-year study, treatment adherence was low in patients with bronchiectasis prescribed inhaled antibiotics for *P. aeruginosa* infection*.* The reported MPR adherence rates of 53% for inhaled antibiotics and other respiratory medicines and 41% for airway clearance (based on self-report) are comparable to studies in asthma, COPD and CF [6-8] but lower than previously reported in bronchiectasis [11,16]. MPR is a more reliable method of adherence measurement than self-report in observational studies conducted in a ‘real-life’ setting [13]; however, it still has the potential to over-estimate adherence [8], making these findings even more clinically concerning. At present, treatment adherence is not routinely assessed in bronchiectasis. We have demonstrated that it is possible to collect these data within a ‘real-life’ setting and are aware that some centres already have access to pharmacy claims data; therefore, potential methods of objectively monitoring adherence in clinical practice are on the horizon. With the prescription of new and potentially expensive therapies for bronchiectasis, particularly for those infected with *P. aeruginosa*[16,17], monitoring of adherence is likely to become an increasingly important aspect of bronchiectasis management.

We studied adherence to three different treatments and found that these treatments had different effects on the health outcomes studied. Similar to findings of other longitudinal adherence studies in CF and COPD [8,12], participants who were adherent to inhaled antibiotics had fewer pulmonary exacerbations, with nearly twice as many exacerbations in the non-adherent group. This is a significant finding given that exacerbations are thought to be associated with disease progression in this population [1]. Whilst the current study did not aim to determine the efficacy of inhaled antibiotics, it did indicate that inhaled antibiotics may be effective in reducing pulmonary exacerbations in bronchiectasis when ≥80% of prescribed medication is consumed. These findings, taken together with the recently reported colistin findings, have important implications for future inhaled antibiotics studies and for the monitoring of adherence to inhaled antibiotics in clinical practice [16]. We cannot discern the direction of the relationship between adherence and pulmonary exacerbations from our data and further research to better understand this relationship is needed. Approximately half of our participants were non-adherent to inhaled antibiotics. Therefore, irrespective of the direction of this relationship, interventions to improve adherence are likely to reduce the frequency of these critical pulmonary events and their associated costs.

The lack of association between adherence and FEV_1_ % predicted is not surprising in this population, given the growing evidence that FEV_1_ is not a sensitive measurement of lung function in bronchiectasis [1,30]. Furthermore, the effectiveness of the treatments included in this study on maintaining or reducing FEV_1_ decline is not known.

Our mean QOL-B domain scores were all lower than those reported in the recent QOL-B paper, particularly for Physical and Role Functioning and Treatment Burden [28]. Inhaler preparations of antibiotics delivered via inhaler rather than nebuliser (e.g. ciprofloxacin dry powder inhaler [17]) may offer a potential method of reducing treatment burden in these patients with bronchiectasis. Inhaler delivery of antibiotics may improve adherence for those in whom time and effort are barriers to treatment. However, it is well recognised that adherence to inhalers in COPD and asthma is approximately 50% in most studies [6,7]. For others, such as those worried about long term use of antibiotics, an inhaler preparation of antibiotics will not be sufficient to overcome these barriers to adherence.

Our results demonstrated that adherence to airway clearance was associated with a higher burden of treatment and worse respiratory symptoms as measured by QOL-B. These findings may appear counter-intuitive when compared to findings in CF [9]; however, the Treatment Burden domain asks patients about the amount of time they commit to their treatments on a daily basis; therefore, those who adherent are likely to spend longer on treatment and may report a higher treatment burden. The relationship between perceived symptoms and adherence is not clear-cut and there are many papers reporting conflicting results [6,8,12,15]. We have recently reported that a lack of belief about necessity for treatment can influence adherence decision-making for airway clearance, particularly when patients do not experience troublesome symptoms [31]. Thus, it could be that this relationship reflects this finding, in that those with more symptoms may be more likely to adhere.

We did not find any association between adherence to inhaled antibiotics or other respiratory medicines and the QOL-B domains studied. This is similar to results from studies exploring associations between adherence to treatment and HRQoL in other chronic respiratory disease populations which have reported conflicting results [6,8,12,15]. The inconsistent effects of medications on HRQoL of patients with bronchiectasis may go some way to explaining this finding. Some medications such as colistin and azithromycin are associated with better HRQoL [16,32] whilst no significant improvement in HRQoL was reported for erythromycin [33]. In addition, the composite nature of the MPR value for ‘other respiratory medicines’ may also have masked any relationships between individual medications (e.g. azithromycin) and QOL-B.

We have recently reported that adherence decision-making in bronchiectasis involves patients weighing up the pros and cons of adhering to treatment [31]. The prescription of medications ‘off-label’ and without proven efficacy may be influencing this decision-making process, as patients may not perceive there to be any benefits of the prescribed treatments [31]. This may be particularly true for inhaled corticosteroids, which are generally not recommended for prescription for bronchiectasis [5] but which were prescribed for 87% of the participants in this study. Thus, there is an urgent need for efficacy studies in this population so that as we begin to focus on improving adherence, we do so for efficacious treatments.

This study was based in a ‘real life’ setting and the cost and lack of availability of electronic monitoring in clinical practice plus its potential behaviour altering effect precluded its inclusion in this study. Therefore, MPRs offered an accurate and feasible method of measuring medication adherence [13]. It is accepted that MPR may overestimate medication adherence as it does not confirm ingestion or inhalation [8]. Carryover and leftover were not included in our MPR calculations; however, the duration of the follow-up period of our study may have reduced the effect of any carryover/left-over as this is likely to average out over time [22]. We collected MPR data on a 6 monthly basis; however, we only calculated MPR over the year of the study rather than 6 monthly to reduce the effect that seasonal variation in symptoms might have on adherence and health outcomes. A cut-off of 80% to denote adherence and non-adherence is commonly used in the adherence literature and in landmark bronchiectasis clinical trials [17,20]. Furthermore, the 80% cut-off has been shown to offer the optimal balance between specificity and sensitivity for self-reported and prescription refill adherence measures [19].

The primary limitation of this study was its sample size. We did not reach the target of 100 participants within the study sites and timeframe. However, the study was not underpowered to detect a difference in pulmonary exacerbations and the sample size is similar to recently reported studies in bronchiectasis [32,33]. The study analysed the associations between adherence data collected over one year with health outcome data collected at one data collection point (12 months). This did not allow us to explore the variability within adherence during the study or to analyse the effect of adherence on outcomes at other time points e.g. 6 months after baseline. In most cases, participants used a regular community pharmacy to dispense all of their medications for bronchiectasis; however, it is possible that some data may have been missing if a participant or carer used a different pharmacy to those contacted. We minimised this by asking participants to provide an up-to-date list at each visit of all of the pharmacies they used and the researcher contacted all of these pharmacies. This study only included patients infected with *P. aeruginosa*, who constitute approximately 20% of the bronchiectasis population [2]. These patients tend to be sicker than other patients with bronchiectasis [3,4]. However, the mean age of 64 years, predominance of women, and the moderate impairment in lung function suggested that our sample was representative of the bronchiectasis population [2].

## Conclusions

This is the first study to demonstrate that adherence is low in bronchiectasis and that it affects important health outcomes including pulmonary exacerbations. Therefore, adherence assessment and monitoring should be considered an integral part of the management of patients with bronchiectasis to ensure that treatment interventions are optimised. Future research should explore reasons for non-adherence and evaluate bronchiectasis-specific strategies to enhance adherence in these patients.

## Abbreviations

BNF: British National Formulary; CF: Cystic fibrosis; COPD: Chronic obstructive pulmonary disease; FEV_1_: Forced expiratory volume in 1 second; HRQoL: Health-related quality of life; IV: Intravenous; MPR: Medication possession ratio; QOL-B: Quality of Life Questionnaire-Bronchiectasis; UK: United Kingdom; US: United States.

## Competing interests

AMcC, MT, JB and CH have no conflicts of interest. AQ has received investigator-initiated grants from Novartis and Gilead Sciences, consulting for Bayer-Schering and Abbott and serves on the North American Scientific Advisory Group to analyse data from ESCF supported by Genentech. SE has received consultancy fees paid to Queen’s University Belfast by Gilead Sciences, Novartis and Forest.

## Authors information

Judy M Bradley and Carmel M Hughes, Joint senior authors.

## Authors’ contributions

AMcC, MT, SE, JB and CH contributed to the conception, design, data collection, analysis and interpretation. AMcC, MT, AQ, SE, JB and CH contributed to the drafting and revision of the manuscript and approval of the final manuscript. All authors read and approved the final manuscript.

## Pre-publication history

The pre-publication history for this paper can be accessed here:

http://www.biomedcentral.com/1471-2466/14/107/prepub

## Supplementary Material

Additional file 1 Treatment adherence and health outcomes in patients with bronchiectasis. Click here for file
