# Supplementary material for: Treatment adherence and health outcomes in patients with bronchiectasis
Source: BMC Pulm Med. 2014 Jul 1;14:107. doi: 10.1186/1471-2466-14-107 (PMC4090650; doi:10.1186/1471-2466-14-107)
Supplement: Additional file 1 — Treatment adherence and health outcomes in patients with bronchiectasis. [file 1471-2466-14-107-S1.pdf]

Treatment adherence and health outcomes in patients with bronchiectasis: a one-year prospective study

Amanda R. McCullough, Michael M. Tunney, Alexandra L. Quittner, J. Stuart Elborn, Judy M. Bradley*, Carmel M. Hughes*.

*Joint senior authors

Additional file 1

Belfast Health and Social Care Trust

Participant Information Sheet

Study Title: Adherence to treatment in non-Cystic Fibrosis (CF) bronchiectasis patients colonised with *Pseudomonas aeruginosa.*

You are being invited to take part in a research study. Before you decide whether or not to take part, it is important that you understand why this research is being completed and what you will be asked to do. Please take time to read the following information and do not hesitate to ask questions about anything that might not be clear to you. Contact details for the researcher can be found at the end of this information sheet. Please take time to decide whether you would or would not like to take part in the study.

What is the purpose of this study?

Bronchiectasis is a chronic lung condition which affects many people in Northern Ireland. It can make people feel short of breath, have a regular cough and produce more phlegm. Some patients with bronchiectasis get infections in their lungs caused by the bacteria (bug) *Pseudomonas aeruginosa*. These infections are usually treated with antibiotics which are sometimes taken with a nebuliser. Patients may also have flare-ups of the *Pseudomonas aeruginosa* infection when their condition is worse which may need to be treated using antibiotic tablets at home or by going into hospital to have antibiotics.

The aim of the study is to find out if taking medication as prescribed affects how often patients who have bronchiectasis and have *Pseudomonas aeruginosa* infection have flare-ups in their condition.

Why have you been chosen?

All patients attending the Chest Clinic who have bronchiectasis and have *Pseudomonas aeruginosa* colonisation are being asked to consider participation in this study.

Do you have to take part?

It is your decision whether or not you would like to take part in the study. You should read this information sheet and ask the researcher to

answer any questions that you have. You will be asked to sign a consent form to show that you have decided to take part. You will be given a copy of this consent form. Your decision whether or not to take part will not affect your medical care. If you decide to take part, you can withdraw from the study at any stage. You are not required to give a reason for your withdrawal and it will not affect your normal care.

What will happen if you take part?

The researcher will call you a minimum of 3 days after you receive this information sheet to arrange a time which suits you to attend the hospital. You will be asked to complete eight short questionnaires which will take between 30 to 45 minutes. You will be asked to do this three times during 12 months; once at the beginning, then after 6 months and again after 12 months. If you are unable to attend the hospital, the researcher may be able to visit you at home at a time convenient to you. At each of these visits, the researcher will carry out a lung function test similar to those done in Chest Clinic and ask you about any techniques you use to help clear the phlegm from your lungs. The researcher will also give you a diary and ask you to keep a record of all the times when you have to take new respiratory medicines or are admitted to hospital with a chest infection. The researcher will phone you at 3 months and again at 9 months after your first visit to collect this information from you and to ask you 3 questionnaires which will take approximately 10 minutes.

The researcher will ask for your permission to take details from your medical notes including physiotherapy and nursing notes for the purposes of the study. If any information needed for the study cannot be taken from your medical notes, then the researcher may ask you about it. You will be asked for the details of your GP and hospital/community pharmacist. The researcher will contact your hospital/community pharmacist to ask them for details of your medication. Your GP will be contacted to tell them that you are taking part in the study.

If you attend the hospital for any of the study visits then you will receive reimbursement of you expenses in line with the Belfast Health and Social Care Trust policy.

Are there any risks or disadvantages of taking part in the study?

There is little risk in taking part in the study and you can withdraw at any time. The questionnaires which you fill in may ask you to think about upsetting

aspects of your disease. If you find this distressing you may withdraw any time. To make it easier for you to participate, questionnaires will be filled in during a time that suits you. Also, if you are unable to attend the hospital to complete questionnaires then the researcher may be able to visit you at home to do this.

What are the benefits of taking part in the study?

By taking part in this study you would be providing information which would help us find out if bronchiectasis patients take their medications as prescribed and whether this affects the number of flare-ups which they have.

What will happen if you decide you no longer wish to take part?

You are free to withdraw from the study at any stage. If you decide to do so, the information recorded until the time you left the study may still be included in the study. Your normal medical care will not be affected if you decide you no longer wish to take part.

Will your details be kept confidential?

All information collected as part of the study will be treated in a confidential manner. Questionnaires and reports will be anonymised. Your name will not appear in any publications. Information gained from the study including patient identifiable information such as consent forms and case report forms will be stored securely at the School of Pharmacy, Queen's University Belfast. It will be kept for five years and then destroyed. This is in line with the Data Protection Act (1998).

The details that you provide as part of this study will be used to contact you in the future about a follow-up study which aims to get more in-depth patient views on treatments in bronchiectasis as well as the things which make it difficult for patients to carry out their treatments. Some of the information you provide as part of this study may be used to determine your eligibility to participate in the follow-up study. If you do not wish to be contacted about the follow-up study please inform the researcher.

What will happen to the results of the research?

The results from the research will be used as part of a PhD project at Queen's University Belfast. They may be published in medical journals and

presented at conferences. All results will be anonymous. You will be provided with a report of the results at the end of the study.

Who is organising and funding the research?

This research is being organised as part of a PhD project at the School of Pharmacy, Queen's University Belfast. It is funded by the Centre for Health Improvement, Queen's University Belfast.

Who has reviewed the study?

The study has received approval from the Office for Research Ethics Committees in Northern Ireland. The project has been peer reviewed by Dr Màire Drain and Dr Susan Martin at the Respiratory Research Office, Belfast City Hospital.

Who can you contact for further information?

Please do not hesitate to contact the researchers as detailed below:

Amanda McCullough
Clinical and Practice Research Group
School of Pharmacy
Queen's University Belfast
Medical Biology Centre
97 Lisburn Road
Belfast
Tel: 02890 972353
Email: amccullough28@qub.ac.uk

Professor Stuart Elborn
Respiratory Consultant
Regional Respiratory Centre
Belfast City Hospital
Lisburn Road
Belfast
BT9 7AB
Tel: 02890263064 **Email:** stuart.elborn@belfasttrust.hscni.net

METHODS

Psychometric properties

Modified Self-reported Medication-taking Scale

The Modified Self-reported Medication-taking scale (e-Table 1) has been validated for use in asthma [1], was used to measure self-reported adherence in this study. It was modified to include an additional question for this study. The addition of this question did not alter the internal validity of this questionnaire (Cronbach's alpha score ≥0·7, with exception of inhaled antibiotics [score 0·5]).

Quality of Life Questionnaire-Bronchiectasis (QOL-B)

The QOL-B is the first bronchiectasis-specific health-related quality of life measure. A paper has recently been published which outlines the development and psychometric properties of this patient reported outcome measure [2]. The QOL-B (version 2.0) has been shown to have excellent psychometric properties, based on data recorded from 89 patients with bronchiectasis (82% infected with *P. aeruginosa*) treated with aztreonam for inhalation solution [2]. The questionnaire has demonstrated good internal consistency (Cronbach's alpha scores ≥0.70), strong test-retest reliability (intraclass correlation coefficients 0.67-0.88) and responsiveness to treatment [2]. Furthermore, the Respiratory Symptoms and Physical Functioning domains correlated with the St George's Respiratory Questionnaire Symptoms ($r = -0.73$, $p<0.001$) and Activity scores ($r = -0.85$, $p<0.001$), respectively and the Physical Functioning domain also correlated with the 6-Minute Walk Test ($r= 0.45$, $p<0.001$) [2].

e-Table 1. Modified Self-reported Medication-taking Scale

Questions
During the last 3 months, have you ever forgotten to take/do[a] your inhaled antibiotics/other respiratory medicines/airway clearance[a]?
During the last 3 months, have you been careless at times about taking/doing[a] your inhaled antibiotics/other respiratory medicines/airway clearance[a]?
During the last 3 months, have you ever stopped taking/doing[a] your inhaled antibiotics/other respiratory medicines/airway clearance[a] because you felt better?
During the last 3 months, have you ever stopped taking/doing[a] your inhaled antibiotics/other respiratory medicines/airway clearance[a] because you felt worse?
During the last 3 months, have you ever stopped taking/doing[a] your inhaled antibiotics/other respiratory medicines/airway clearance[a] on time and regularly?[b]

[a]individual questionnaires were used for each treatment type
[b]additional question

REFERENCES

1. Brooks CM, Richards JM, Kohler CL, Soong SJ, Martin B, Windsor RA, Bailey WC: **Assessing adherence to asthma medication and inhaler regimens: a psychometric analysis of adult self-report.** *Med Care* 1994, **32**(3):298–307.

2. Quittner AL, Marciel KK, Salathe MA, O'Donnell AE, Gotfried MH, Ilowite JS, Metersky ML, Flume PA, Lewis SA, McKevitt M, Montgomery AB, O'Riordan TG, Barker AF**: A preliminary Quality of Life Questionnaire-Bronchiectasis: a patient-reported outcome measure for bronchiectasis**. *Chest* 2014; doi:10.1378/chest.13-1891
